# Effects of the type and quality of usual source of care on medical expenditures in adults with diabetes before and during the COVID‑19 pandemic: a panel data analysis using the Korea Health Panel (2019–2022)

**DOI:** 10.1186/s12913-025-13518-7

**Published:** 2025-10-15

**Authors:** Hyun-Young Shin, Kyoungwoo Kim, Hwa-Young Lee, Jae-Ho Lee

**Affiliations:** 1 Department of Family Medicine, Seoul St. Mary’s Hospital, College of Medicine, The Catholic University of Korea, 222, Banpo-daero, Seocho-gu, Seoul, 06591 Republic of Korea; 2 Department of Family Medicine, Inje University Haeundae Paik Hospital, Busan, Republic of Korea; 3 Graduate School of Public Health and Healthcare Management, The Catholic University of Korea, Seoul, Republic of Korea; 4 Catholic Institute for Public Health and Healthcare Management, The Catholic University of Korea, Seoul, Republic of Korea

**Keywords:** Diabetes, Usual source of care, Primary care, Medical expenditures, COVID-19 pandemic, Panel data analysis

## Abstract

**Background:**

South Korea experiences the highest diabetes-related hospitalization rates among OECD countries. Although integrated primary care could potentially lower preventable hospitalizations and healthcare costs, the limited uptake of a usual source of care (USC) and underdeveloped primary care services in Korea have impeded progress, and the role and functions of primary care remain insufficiently defined.

**Methods:**

This study investigated how having a USC affects medical expenditures in adults with diabetes by using Korea Health Panel data from 2019 to 2022, spanning the pre-pandemic (2019) and pandemic (2020–2022) periods. The analysis included 6,144 individuals. The main independent variable was the type and quality of USC, categorized into three groups: no USC, place-only USC, and physician-based USC. Quality was assessed by patient-reported comprehensiveness and coordination, combined into an integrated quality index. We applied panel regression analysis using the Hausman–Taylor estimation technique to address both time-varying and time-invariant covariates while mitigating endogeneity concerns.

**Results:**

Between 2019 and 2022, the proportion of individuals with a physician at a regular location serving as their USC increased from 58.5% to 66.1%, while the proportion without a physician as their USC decreased from 15.1% to 10.9%, with larger variation among vulnerable populations. The physician-based USC group consistently incurred higher absolute expenditures but showed the smallest relative increase during the pandemic, compared with sharp rises in the no-USC and place-only USC groups. Adults with diabetes whose USC was a high-quality primary care physician—defined by favorable patient ratings of comprehensiveness and coordination—were associated with approximately 13.1% lower healthcare expenditures compared to those lacking a physician as their USC (*β* = − 0.140, *P* = 0.006). Moreover, community clinic–based physicians as USC were associated with lower expenditures, while hospital-based USC was associated with higher costs, though not statistically significant.

**Conclusion:**

These findings underscore the importance of increasing access to high-quality primary care physicians as USC to optimize chronic disease management and maintain sustainable healthcare systems.

**Supplementary Information:**

The online version contains supplementary material available at 10.1186/s12913-025-13518-7.

## Introduction

Primary care is characterized by four essential attributes—first-contact access, continuity, comprehensiveness, and coordination—delivered in a person-focused manner and oriented to families and communities [1]. A usual source of care (USC) refers to a regular physician, clinic, or health center that individuals usually visit for advice or treatment, excluding hospital emergency departments [2]. Having a USC is considered a structural prerequisite for realizing the core functions of primary care: it facilitates first-contact access, supports continuity, and strengthens comprehensiveness and coordination. Accordingly, the presence of a USC has been associated with multiple health benefits, including improved access to services, greater uptake of preventive care, enhanced chronic disease management, and reduced unmet medical needs [1, 3]. These benefits are particularly critical for diabetes care, where consistent management and integrated services are essential to improving outcomes and controlling healthcare costs.

In the context of South Korea’s healthcare system, primary care remains relatively weak within a predominantly hospital-centered delivery model that allows patients to bypass clinics and directly access secondary or tertiary hospitals. Previous research has highlighted structural limitations such as fragmented services, under-resourced community clinics, and the absence of formal care coordination mechanisms [4]. Similarly, OECD data indicate that Korea performs below the OECD average on several primary care performance indicators, including avoidable hospitalization rates, underscoring the need to strengthen first-contact and continuous care [5].

Globally, reinforcing primary care is considered a principal approach to meet the demands generated by aging societies and the escalating prevalence of chronic diseases [6]. Diabetes, a typical ambulatory care–sensitive condition (i.e., one for which effective outpatient care can reduce the risk of hospitalization), represents a core metric for assessing primary care efficacy through analysis of potentially preventable hospitalization rates [7, 8]. The Republic of Korea (hereafter Korea) continues to report exceptionally high diabetes-related hospitalization rates among OECD member states [5]. In 2021, Korea had the highest age-standardized rate of diabetes-related disability-adjusted life years (DALYs) among high-income countries, at 966.4 per 100,000 people [9]. This phenomenon accompanies the rapid aging of the population, with those aged 65 years and older now making up more than 20% of the population, further contributing to rising medical expenditures [10]. These obstacles reveal persistent structural limitations in Korea’s primary care, including disjointed chronic disease management, suboptimal infrastructure for preventive care, and insufficient patient educational resources [11, 12].

Effective diabetes management in primary care settings has the potential to decrease unnecessary hospitalizations and lower associated healthcare expenditures. A Korean study demonstrated that having a usual source of care (USC) with a designated physician was associated with higher medication adherence among diabetes patients, whereas a place-only USC did not yield similar benefits [13]. Expanding international research indicates that continuity of care with a USC is linked to higher rates of preventive service uptake, improved chronic disease outcomes, and decreased overall medical costs [14–16]. In particular, longitudinal relationships with a USC have resulted in fewer emergency department visits and hospitalizations in diverse populations [14, 16]. For diabetes patients, maintaining continuity with a USC has been associated with better glycemic control, fewer diabetes-related complications, and lower healthcare expenditures [17–19].

To improve chronic disease management in primary care, numerous high-income countries have implemented pay-for-performance (P4P) incentive schemes [20]. Systematic reviews have demonstrated that such financial incentives are associated with significant reductions in diabetes-related hospitalizations and mortality [21]. Additional analyses emphasize that having a regular primary care provider and achieving glycemic control are crucial elements in decreasing hospitalization rates for diabetes patients; these studies underscore that high levels of continuity and integrated care delivery are fundamental to achieving favorable health outcomes [7]. However, current evidence of the cost-effectiveness of diabetes care within the Patient-Centered Medical Home (PCMH) model remains limited and inconclusive [22]. Established strategies supported by evidence include strengthening patient education and self-management support, deploying team-based care models to optimize care coordination, and leveraging electronic patient registries for population health management [23].

The COVID-19 pandemic revealed significant weaknesses in the management of chronic diseases, particularly for individuals with diabetes, as a result of healthcare facility closures and decreased access to medical services. While the significance of continuous care with a USC has been widely acknowledged, research focused on its role in chronic disease management in Korea—especially concerning trends in medical expenditures and the impact of the pandemic—remains scarce. In light of these challenges, Korea’s newly established administration, which assumed office in June 2025, has committed to launching a national primary care physician (PCP) program. This initiative is designed to formalize the USC framework, with the goal of enhancing chronic disease management and resolving persistent problems related to continuity of care within the Korean healthcare system. While the program details remain under development, it is expected to be voluntary and to provide financial incentives for patients who register with a community-based primary care physician. Unlike the current system, where clinic physicians play little gatekeeping role, the envisioned program aims to strengthen both gatekeeping and coordination functions, thereby expanding the role of primary care in Korea’s healthcare delivery.

In this context, this study employs longitudinal data from the Korea Health Panel (2019–2022) to investigate how the availability and quality of USC influence medical expenditures among adults with diabetes. By evaluating trends before and during the COVID-19 pandemic, the study aims to provide evidence to guide the development of policies that enhance continuity of care within resource-limited public health systems.

## Methods and materials

### Data source and study population

Data for this research were drawn from the Korea Health Panel (KHP) Survey, a nationally representative longitudinal survey collaboratively administered by the Korea Institute for Health and Social Affairs and the National Health Insurance (NHI) Service [24]. The KHP is an open-access public database that independently collects information on healthcare utilization and medical expenditures at the individual level; the authors did not participate in its data collection. For this study, we used the officially released dataset (KHP version 2.3, 2019–2022) and restricted the sample to adults aged 19 years or older who self-reported a diagnosis of diabetes. The final unbalanced panel consisted of 6,144 person-year observations (*n* = 1,589 in 2019; *n* = 1,520 in 2020; *n* = 1,525 in 2021; *n* = 1,510 in 2022). The outbreak of COVID-19 in South Korea was first reported on January 20, 2020, and on May 1, 2024, COVID-19 was reclassified as a Class 4 notifiable infectious disease. Therefore, in this study, the year 2019 is considered the pre-COVID-19 period, while the period from 2020 onward is regarded as the COVID-19 era for analysis.

### Outcome variable

The main outcome variable was the annual total medical expenditures. In Korea’s NHI system, patients usually pay a statutory co-payment for covered services, while non-covered services are billed at provider discretion and fully paid out-of-pocket by patients. In this analysis, medical expenditure was defined as the aggregate of co-payments for covered services, out-of-pocket expenses for non-covered services, and NHI payments. Expenditures related to dental services were not included in the evaluation. Due to the skewed nature of expenditure data, total medical costs underwent log transformation [25]. Individuals reporting zero medical expenditures were omitted because log transformation is undefined at zero and such cases likely represent non-participation in the healthcare system [26].

### Main independent variable: type of USC

The main explanatory variable was the type of USC, classified into three groups: no USC, having a usual place only, and having a usual physician with a place. Subcategories were further divided by facility type (community clinic vs. hospital) and by the quality of primary care, which was evaluated across three domains: comprehensiveness, coordination, and an integrated quality index. These domains corresponded to the following KHP survey items: (1) “Do you have a regular medical facility (such as a clinic or hospital) or doctor that you usually visit when you are sick or need a medical consultation, examination, or treatment?” (binary response: Yes or No); (2) “Does the physician who serves as your usual source of care deal with almost all of your routine health problems?” (comprehensiveness); and (3) “Does the physician who serves as your usual source of care appropriately refer you to health care facilities or providers needed to manage your health?” (coordination). Items (2) and (3) were measured using 5-point Likert scales. The integrated quality index combined comprehensiveness and coordination functions. High in quality was defined as patient ratings of ‘good’ or ‘very good’ for both domains. Not high in quality was defined as ratings of ‘moderate,’ ‘poor,’ or ‘very poor’ in one or both domains.

### Covariates

Sociodemographic covariates included age group (19–49, 50–64, ≥ 65), sex, education level (elementary, middle/high school, college or above), equivalized personal income quintile, and type of public health insurance (employee, regional, or Medical Aid). Health-related covariates consisted of log-transformed annual healthcare utilization (outpatient, inpatient, and emergency visits), Charlson Comorbidity Index (CCI) score [27] (1 vs. ≥2), and subjective health status (good, moderate, poor).

### Statistical analysis

Descriptive analyses were carried out to evaluate the distribution of USC types and their relationships with sociodemographic and health-related characteristics over multiple survey years. To examine the longitudinal association between USC and total medical expenditures, we compiled a balanced panel of 4,580 individuals who participated in all four waves of the survey. A Hausman–Taylor panel regression model was utilized to estimate the effects, adjusting for both time-varying and time-invariant covariates, as well as potential endogeneity of select regressors [28]. All statistical analyses, including the Hausman–Taylor panel regression, were conducted using the PROC PANEL procedure in SAS software (version 9.4; SAS Institute Inc., Cary, NC, USA).

The panel regression model was constructed as follows:

$$\eqalign{& Ln\left( {total\,medical\,cost_{it}} \right)\> \cr & { =\beta _1}X_{it}^{\left(1 \right)}{ + \beta_2}X_{it}^{\left( 2 \right)}{ + \gamma_1}Z_i^{\left( 1 \right)} + {u_i}{ + \varepsilon_{it}}, \cr} $$

where ln (total medical cost_it_) denotes the log-transformed total medical expenditures for individual i at time t; X_it_^(1)^ and X_it_^(2)^ represent time-varying exogenous variables (e.g., USC type, insurance type, age group) and time-varying endogenous variables (e.g., log-transformed healthcare utilization, subjective health status, CCI score, equivalized personal income quintiles), respectively; Z_i_^(1)^ represents time-invariant exogenous variables (e.g., sex, education level); u_i_ accounts for unobserved individual-specific effects; and ε_it_ denotes the idiosyncratic error term. This model enables unbiased estimation of coefficients for time-invariant regressors—which fixed-effects models typically exclude—while mitigating bias due to correlation between individual-specific effects and endogenous variables. Endogenous regressors were instrumented using internal instruments derived from the exogenous variables, as specified in the Hausman–Taylor methodology [28].

We selected the Hausman–Taylor estimator because it combines the strengths of fixed- and random-effects models: unlike fixed-effects, it allows estimation of time-invariant regressors such as sex and education level, and unlike random-effects, it addresses potential correlation between unobserved individual-specific effects and endogenous regressors. To test robustness, we additionally estimated fixed-effects and random-effects models as sensitivity analyses. While the fixed-effects model did not yield statistically significant results—likely reflecting limited within-individual variation in USC status across four years—the directions of effect estimates were consistent with those from the Hausman–Taylor and random-effects models. These supplementary results are reported in the Appendix, and their implications are further discussed in the Discussion section.

## Results

### Trends in types of USC (Table 1)

**Table 1:** Yearly trends in types of USC and associated health characteristics in adults diagnosed with diabetes

		2019 (*n* = 1,589)			2020 (*n* = 1,520)			2021 (*n* =1,525)			2022 (*n*= 1,510)		
		No USC (*n*=232)	Place only (*n*=401)	Physician with place (*n*=956)	No USC (*n*=358)	Place only (*n*=360)	Physician with place (*n*=802)	No USC (*n*=163)	Place only (*n*=354)	Physician with place (*n*=1,008)	No USC (*n*=143)	Place only (*n*=378)	Physician with place (*n*=989)
		15.1	26.4	58.5	22.3	23.2	54.6	10.6	19.9	69.4	10.9	23.0	66.1
Age (years)	19 – 49	24.6	22.6	52.8	24.4	28.4	47.2	15.2	21.1	63.7	26.9^*^	18.9^*^	54.2^*^
	50 – 64	15.6	25.3	59.2	22.7	20.2	57.1	10.3	19.5	70.2	8.5^*^	24.7^*^	66.9^*^
	65 or older	12.3	28.3	59.4	21.4	23.9	54.7	9.7	20.0	70.3	7.9^*^	22.9^*^	69.1^*^
Sex	Male	15.0	26.9	58.2	20.4	22.9	56.7	10.2	19.8	70.0	11.7	24.0	64.3
	Female	15.2	25.9	58.9	24.4	23.5	52.1	11.1	20.2	69.8	9.7	21.7	68.6
Marital status	Never married	14.9	26.2	58.9	24.5	22.6	52.9	13.1	14.1	72.8	18.5	14.5	67.0
	Married	15.8	25.9	58.3	20.7	23.3	56.0	8.6	21.8	69.6	10.5	23.2	66.3
	Other ^a^	13.5	27.6	58.9	25.3	23.0	51.7	14.7	17.1	68.2	10.2	23.9	65.9
Education	Elementary level or below	13.2	25.5	61.3	22.4	24.8	52.8	11.0	20.2	68.8	8.9^*^	23.2^*^	67.9^*^
	Middle or High school	14.7	29.3	56.0	22.6	22.4	55.0	10.3	19.2	70.5	8.0^*^	24.1^*^	67.9^*^
	College or higher	18.3	21.5	60.3	21.3	22.9	55.7	11.1	21.1	67.8	17.9^*^	20.7^*^	61.4^*^
Equivalized personal income	First	10.8	27.6	61.6	23.2	22.2	54.6	11.5	22.0	66.5	8.5	25.5	66.0
	Second	16.1	22.1	61.8	23.2	25.2	51.6	15.2	18.4	66.4	10.7	24.8	64.5
	Third	14.3	30.3	55.5	28.0	24.3	47.7	9.7	17.7	72.6	8.4	23.9	67.7
	Fourth	12.7	28.2	59.0	20.0	24.6	55.4	3.9	22.6	73.5	12.3	17.0	70.7
	Fifth	23.2	25.4	51.4	17.7	20.2	62.1	11.7	17.8	70.5	14.4	22.3	63.3
Health insurance	Employee	16.2	26.4	57.4	21.7	24.8	53.5	9.3	19.6	71.1	10.5	23.9	65.6
	Regional	14.2	25.9	59.9	24.1	17.9	58.0	14.1	20.0	65.9	11.6	20.9	67.5
	Medical Aid	9.2	28.9	61.9	18.7	32.3	49.0	7.0	23.0	70.0	10.8	24.2	64.9
Subjective health status	Good	19.1	25.3	55.6	21.1	21.1	57.8	11.4	17.0	71.6	12.1	24.1	63.8
	Moderate	13.7	26.5	59.8	24.5	21.6	53.9	10.8	21.5	67.8	10.5	22.0	67.5
	Poor	13.9	27.3	58.8	19.7	26.5	53.7	9.9	19.7	70.3	10.7	23.8	65.5
CCI score	1	15.6	26.1	59.3	22.4^*^	22.2^*^	55.4^*^	11.2^*^	19.7^*^	69.0^*^	11.3	22.7	66.0
	2 or more	7.8	30.8	61.4	19.7^*^	37.7^*^	42.6^*^	2.5^*^	22.9^*^	74.6^*^	4.6	27.1	68.3

Data source: KHP (2019–2022). Results are shown as weighted percentages; due to rounding, row totals may not equal exactly 100%. The Rao–Scott Chi-square test assessed group differences. ^a^ Refers to individuals who are divorced, separated, or widowed. ^*^ *P* < 0.05. KHP: Korea Health Panel; USC: usual source of care; CCI: Charlson Comorbidity Index

Between 2019 and 2022, the proportion of Korean adults (≥ 19 years) with diabetes indicating a physician at a regular place as their USC rose from 58.5% to 66.1%, while the proportion without any USC decreased from 15.1% to 10.9%. There was a temporary drop in physician-based USC in 2020, with a subsequent recovery observed by 2022. By 2022, adults aged ≥ 65 years had the highest rate of physician-based USC (69.1%), while those with a college degree or higher had the lowest (61.4%). Although most sociodemographic subgroups remained relatively stable over time, vulnerable populations experienced significant disruptions during the initial pandemic phase: from 2019 to 2020, the percentage without any USC increased notably among older adults (12.3% to 22.4%), individuals with less education (13.2% to 21.8%), those in the lowest income quintile (10.8% to 23.2%), Medical Aid recipients (9.2% to 18.7%), and those with higher comorbidity (CCI ≥ 2; 7.8% to 19.7%).

### Annual trends in healthcare utilization and costs by USC type (Table 2-a & 2-b)

**Table 2a:** Yearly patterns in healthcare expenditures by USC type among adults with diabetes

	2019				2020				2021				2022			
	No USC	Place only	Physician with place	Mean	No USC	Place only	Physician with place	Mean	No USC	Place only	Physician with place	Mean	No USC	Place only	Physician with place	Mean
	*n* = 232	*n* = 401	*n* = 956	*n* = 1,589	*n* = 358	*n* = 360	*n* = 802	*n* = 1,520	*n* = 163	*n* = 354	*n* = 1,008	*n* = 1,525	*n* = 143	*n* = 378	*n* = 989	*n* = 1,510
Total medical expenditure (1,000 KRW)	1,145 ± 164	1,485 ± 228	2,047 ± 180	1,762 ± 125	1,779 ± 255	2,013 ± 250	2,122 ± 212	2,020 ± 140	1,269 ± 198	2,065 ± 283	2,123 ± 173	2,021 ± 134	1,689 ± 344	2,821 ± 349	2,386 ± 188	2,411 ± 153
OOP cost covered by the NHI (1,000 KRW)	214 ± 27	277 ± 35	314 ± 22	289 ± 16	331 ± 29	291 ± 29	312 ± 25	311 ± 17	275 ± 40	336 ± 40	318 ± 19	317 ± 16	351 ± 67	426 ± 38	388 ± 26	393 ± 21
OOP cost not covered by the NHI (1,000 KRW)	187 ±38	191 ± 31	241 ± 33	219 ± 22	311 ± 75	228 ± 43	249 ± 29	258 ± 25	284 ± 80	279 ± 52	230 ± 13	246 ± 20	299 ± 100	303 ± 42	278 ± 29	286 ± 24
NHI payments (1,000 KRW)	744 ± 120	1,017 ± 182	1,492 ± 149	1,254 ± 102	1,138 ± 163	1,495 ± 219	1,561 ± 183	1,452 ± 117	710 ± 117	1,450 ± 220	1,575 ± 156	1,458 ± 116	1,040 ± 239	2,091 ± 304	1,720 ± 161	1,731 ± 131

Data source: KHP (2019–2022). Values are shown as weighted mean ± standard error (SE). The ANOVA test was utilized to compare weighted means. All Ps ≥ 0.05. USC: usual source of care; KHP: Korea Health Panel; NHI: National Health Insurance; OOP: out-of-pocket

**Table 2b:** Yearly patterns in healthcare utilization by USC type among adults with diabetes

	2019				2020				2021				2022			
	No USC	Place only	Physician with place	Mean	No USC	Place only	Physician with place	Mean	No USC	Place only	Physician with place	Mean	No USC	Place only	Physician with place	Mean
	*n* = 232	*n* = 401	*n*=956	*n* = 1,589	*n* = 358	*n* = 360	*n* = 802	*n* = 1,520	*n* = 163	*n* = 354	*n* = 1,008	*n* = 1,525	*n* = 143	*n* = 378	*n* = 989	*n* = 1,510
Total episodes of healthcare utilization	23.70^*^ ± 1.79	25.60^*^ ± 1.14	28.31^*^ ± 1.00	26.90 ± 0.72	25.51^*^ ± 1.29	25.25^*^ ± 1.30	28.38^*^ ± 1.10	27.02 ± 0.73	26.28 ± 2.66	29.29 ± 1.50	30.81 ± 1.03	30.02 ± 0.82	23.64^*^ ± 2.08	30.11^*^ ± 1.45	30.11^*^ ± 1.00	29.41 ± 0.77
Outpatient care	23.40^*^ ± 1.78	25.33^*^ ± 1.13	27.91^*^ ± 1.00	26.55 ± 0.71	25.17^*^ ± 1.27	24.88^*^ ± 1.28	27.98^*^ ± 1.10	26.64 ± 0.72	26.00 ± 2.65	28.93 ± 1.48	30.37 ± 1.01	29.62 ± 0.81	23.28^*^ ± 2.07	29.61^*^ ± 1.43	29.54^*^ ± 0.98	28.88 ± 0.76
Inpatient care	0.17 ± 0.03	0.20 ± 0.03	0.29 ± 0.04	0.25 ± 0.02	0.24 ± 0.04	0.23 ± 0.03	0.28 ± 0.03	0.26 ± 0.02	0.23 ± 0.06	0.21 ± 0.03	0.26 ± 0.03	0.25 ± 0.02	0.27 ± 0.06	0.36 ± 0.04	0.40 ± 0.08	0.38 ± 0.05
ED visit	0.12 ± 0.03	0.07 ± 0.02	0.10 ± 0.01	0.10 ± 0.01	0.10 ± 0.02	0.14 ± 0.03	0.13 ± 0.02	0.12 ± 0.01	0.10 ± 0.06	0.15 ± 0.04	0.17 ± 0.02	0.16 ± 0.02	0.10 ± 0.04	0.14 ± 0.02	0.17 ± 0.02	0.15 ± 0.01

Data source: KHP (2019–2022). Values are shown as weighted mean ± standard error (SE). The ANOVA test was utilized to compare weighted means. ^*^ *P* < 0.05. USC: usual source of care; KHP: Korea Health Panel; ED: emergency department

Individuals with a physician-based USC consistently incurred higher total medical expenditures and more frequent healthcare utilization compared to those with no USC or a place-only USC. In 2022, the physician-based USC group had mean total medical expenditures of 2.39 million KRW, compared to 1.69 million KRW among the no-USC group. Annual per capita expenditures increased from 1.76 million KRW in 2019 to 2.41 million in 2022, with the most substantial increase (55.4%) between 2019 and 2020 observed in individuals without any USC. Rates of healthcare utilization, especially for outpatient visits, remained significantly greater in the physician-based USC group throughout the observed years, whereas inpatient and emergency department use did not display consistent patterns between USC types. The average number of visits per person increased modestly from 2019 (26.9) to 2022 (29.4): the physician-based group showed a rise from 28.3 to 30.1, while the no-USC group decreased from 23.7 to 23.6.

### Quality of care by institution type (Table 3)

**Table 3:** Comprehensive care and coordination roles of physicians serving as USC for adults with diabetes

		Community clinic (primary care practice)				Hospital			
		2019	2020	2021	2022	2019	2020	2021	2022
		(*n* = 542)	(*n* = 514)	(*n* = 650)	(*n* = 683)	(*n* = 395)	(*n* = 278)	(*n* = 350)	(*n* = 297)
Comprehensiveness	Very good	88 (16.6)	105 (25.0)	99 (17.4)	130 (17.8)	88 (24.0)	47 (17.8)	42 (11.6)	54 (17.6)
	Good	329 (58.6)	315 (56.2)	417 (64.4)	422 (62.3)	223 (52.7)	176 (63.1)	229 (67.3)	189 (58.3)
	Moderate	82 (13.9)	71 (13.9)	107 (15.1)	100 (17.3)	48 (14.1)	37 (11.0)	57 (14.5)	35 (14.6)
	Poor	31 (6.8)	18 (3.9)	25 (2.6)	30 (2.5)	34 (8.8)	16 (6.7)	20 (5.9)	14 (8.6)
	Very poor	12 (4.0)	5 (1.1)	2 (0.5)	1 (0.1)	2 (0.4)	2 (1.5)	2 (0.6)	5 (0.9)
Coordination of care	Very good	52 (9.2)	64 (17.1)	70 (13.9)	64 (6.7)	41 (10.5)	18 (7.1)	22 (5.1)	34 (10.4)
	Good	161 (29.7)	144 (28.4)	232 (33.4)	278 (41.4)	113 (25.1)	92 (36.9)	121 (32.3)	129 (40.7)
	Moderate	96 (14.7)	140 (23.5)	166 (26.4)	171 (25.7)	76 (15.0)	83 (27.6)	110 (33.4)	70 (24.6)
	Poor	154 (29.2)	131 (24.6)	131 (18.1)	115 (19.1)	104 (29.0)	67 (21.7)	68 (20.6)	47 (18.5)
	Very poor	79 (17.2)	35 (6.3)	51 (8.1)	55 (7.1)	61 (20.4)	18 (6.7)	29 (8.6)	17 (5.9)

Data source: KHP (2019–2022). Population: adults aged ≥19 years with diabetes mellitus. Values are displayed as frequency and weighted percentage; column total = 100%. Public health centers and herbal medical institutions were omitted due to small sample sizes (fewer than 10 respondents per year). The following questions were employed to evaluate each attribute: Comprehensiveness — “Does the physician serving as your usual source of care manage nearly all of your routine health concerns?”; Coordination — “Does the physician serving as your usual source of care ensure proper referrals to other providers or facilities as necessary?”

USC: usual source of care; KHP: Korea Health Panel

For those with a physician-based USC, providers affiliated with clinics were rated marginally higher than hospital-affiliated providers regarding comprehensiveness and coordination of care. In 2020, 81.2% of clinic-based physicians received a rating of “very good” or “good” for comprehensiveness, compared to 80.9% for hospital-based counterparts. For coordination of care, the respective rates were 45.5% and 44.0%. The proportion of USC physicians affiliated with hospitals decreased from 42.2% in 2019 to 30.3% in 2022, as derived from the sample counts shown in Table 3.

### Effects of having a high-quality primary care physician on total medical expenditures (Table 4)

**Table 4:** Impact of high-quality primary care physicians on aggregate medical expenditures among adults with diabetes

			Parameter estimate	SE	t value	*P*
Constant term			9.66	0.248	38.92	< 0.001
Age group (ref: 19–49 years)	50–64 years		0.105	0.133	0.79	0.432
	65 years or older		0.202	0.134	1.51	0.132
Sex (ref: male)	Female		-0.043	0.049	-0.72	0.472
Education level (ref: elementary or less)	Middle to high school		0.028	0.064	0.53	0.593
	College or higher		0.027	0.101	-0.26	0.793
Marital status (ref: never married)	Divorced, widowed, or separated		-0.351	0.181	-1.94	0.052
	Married		-0.388	0.177	-2.20	0.028
Equalized personal income quintile ^a^ (ref: first)	Second		0.137	0.061	2.26	0.024
	Third		0.099	0.081	1.23	0.220
	Fourth		0.085	0.093	0.91	0.363
	Fifth		0.214	0.117	1.83	0.068
Health insurance type (ref: employee)	Regional		0.097	0.051	1.91	0.063
	Medical Aid		0.118	0.107	1.11	0.267
CCI score (ref: 1)	2 or more		0.092	0.171	0.54	0.592
Number of healthcare utilizations (log-transformed)			1.225	0.040	30.80	< 0.001
Subjective health status (ref: good)	Moderate		0.102	0.054	1.87	0.062
	Poor		0.197	0.062	3.16	0.002
Type of physician as a USC (ref: no usual physician)	PCP	High in quality	-0.140	0.051	-2.73	0.006
		Not high in quality	-0.087	0.047	-1.84	0.067
	Hospital physician		0.086	0.048	1.77	0.076
Survey year (ref: 2019)	2020		0.075	0.042	1.78	0.075
	2021		0.026	0.043	0.62	0.537
	2022		0.186	0.044	4.26	< 0.001

Panel regression analysis was conducted using the Hausman–Taylor estimation method in SAS 9.4, based on a balanced panel derived from the KHP (2019–2022). The dependent variable was total medical expenditures in log-transformed form. The key independent variable was having a high-quality PCP as the USC. The definition of “high in quality” was a USC physician whom the patient rated positively (i.e., ‘good’ or ‘very good’) for both comprehensiveness and coordination functions; “not high in quality” corresponded to having either or both domains rated as neutral or negative (i.e., ‘moderate,’ ‘poor,’ or ‘very poor’). The following variables were modeled as endogenous and instrumented in the analysis: CCI, log-transformed healthcare utilization count, subjective health status, and equivalized personal income quintile. Individuals with no healthcare use in the previous year or who indicated a public health center or herbal clinic as their USC were excluded from the study

KHP: Korea Health Panel; USC: usual source of care; PCP: primary care physician; CCI: Charlson Comorbidity Index

Panel regression analysis using Hausman–Taylor estimation indicated that having a high-quality primary care physician—defined as a clinic-based physician rated “very good” or “good” for both comprehensiveness and coordination—was significantly associated with 13.1% lower in total medical expenditures compared to individuals without a physician-based USC (*β* = − 0.140, *P* = 0.006). Having a physician rated lower in quality showed a non-significant trend toward an 8.3% reduction (*β* = − 0.087, *P* = 0.067). Similarly, hospital-based physicians as USC exhibited a non-significant trend toward 9.0% higher expenditures (*β* = 0.086, *P* = 0.076). Increased total healthcare utilization (log-transformed number of episodes) and worse self-reported health status were also significantly related to higher expenditures. The regression model was adjusted for age, sex, marital status, education, equivalized personal income quintiles, insurance type, CCI score, and survey year, with 2019 serving as the reference category.

### Impact of various types and qualities of usual source of care on total medical expenditures (Table 5)

**Table 5:** Impact of varying USC types on aggregate medical expenditures among adults with diabetes

			Estimate	SE	t value	*P*
Having a USC (ref: no USC)	Having a USC		-0.015	0.051	-0.29	0.768
Place designated as a USC (ref: no usual place)	Having a usual place		-0.016	0.050	-0.32	0.749
Physician serving as a USC (ref: no usual physician)	Having a usual physician		-0.036	0.037	-0.98	0.326
Type of USC (ref: no USC)	Having only a usual place		0.011	0.058	0.20	0.844
	Having both a usual physician and usual place		-0.029	0.053	-0.55	0.582
Place designated as a USC (ref: no usual place)	Community clinic		-0.113	0.053	-2.13	0.033
	Hospital		0.128	0.057	2.27	0.023
Physician serving as a USC (ref: no usual physician)	Community clinic physician (i.e., PCP)		-0.109	0.041	-2.64	0.008
	Hospital-based physician		0.086	0.048	1.78	0.075
Usual physician quality (ref: no usual physician)	High		-0.070	0.045	-1.57	0.116
	Not high		-0.013	0.041	-0.32	0.749
Comprehensiveness of PCP as a USC (ref: no usual physician)	PCP	High	-0.140	0.043	-3.24	0.001
		Not high	0.018	0.068	0.27	0.788
	Hospital usual physician		0.085	0.048	1.76	0.079
Coordination function of PCP as a USC (ref: no usual physician)	PCP	High	-0.129	0.051	-2.54	0.011
		Not high	-0.094	0.048	-1.97	0.049
	Hospital usual physician		0.086	0.048	1.78	0.076

Panel regression analyses were conducted utilizing the Hausman–Taylor estimator in SAS 9.4 on a balanced panel dataset derived from the KHP (2019–2022). The dependent variable was the log-transformed value of total medical expenditures. The primary explanatory variables encompassed types and characteristics of USC. Covariates comprised age, sex, marital status, educational attainment, insurance type, CCI score, the log-transformed number of healthcare visits, perceived health status, and equivalized personal income quintile. Endogenous variables (CCI score, number of visits, perceived health status, and income quintile) were instrumented within the analytic model. Subjects who had not used healthcare services in the preceding year or whose USC was listed as a public health center or herbal clinic were excluded. “High in quality” designated a USC physician rated either “good” or “very good” on both: (1) comprehensiveness (“deals with most routine health problems”) and (2) coordination (“refers appropriately to needed care”)

KHP: Korea Health Panel; USC: usual source of care; PCP: primary care physician; CCI: Charlson Comorbidity Index

Extended panel regression analyses were conducted to further investigate how different types and qualities of USC are associated with total medical expenditures among adults with diabetes. Relative to those without a USC, individuals utilizing a community clinic as their USC experienced a 10.7% decrease in expenditures (*β* = − 0.113, *P* = 0.033), whereas those designating a hospital as their USC incurred a 13.7% increase (*β* = 0.128, *P* = 0.023). Utilizing a physician at a community clinic as the USC was linked to a 10.3% reduction in expenditures when compared to those lacking a physician-based USC (*β* = − 0.109, *P* = 0.008). Among those with a clinic-based physician as their USC, participants who rated comprehensiveness as “very good” or “good” reported 13.1% lower expenditures (*β* =–0.140, *P* = 0.001), and those attributing similar ratings for coordination demonstrated a 12.1% reduction (*β* = − 0.129, *P* = 0.011), both compared to counterparts without a physician-based USC.

## Discussion

The analysis revealed that the proportion of adults with diabetes reporting a USC diminished during the initial phase of the COVID-19 pandemic in 2020, but later rebounded in 2021 and exceeded pre-pandemic levels by 2022. This temporary decline was especially marked among high-risk groups—such as those with multiple comorbidities, poor perceived health, limited education and income, and recipients of Medical Aid—indicating their greater vulnerability to disruptions in primary care access. Adults with diabetes who had consistent, high-quality primary care physicians were associated with approximately 13.1% lower overall medical expenditures, whereas other categories (e.g., lower-rated or hospital-based USC) showed non-significant trends. These results underscore the critical role of sustained primary care access, mediated by broader social determinants of health (SDOH), in supporting diabetes management. Key SDOH variables, including access to healthcare, physical environment, socioeconomic factors (income, education, insurance), care quality, food insecurity, and health-related behaviors, have strong correlations with health outcomes among diabetes patients [29, 30]. In one U.S. study, each additional point in SDOH score corresponded to a 33% increased risk of all-cause mortality [31].

The COVID-19 pandemic intensified existing disparities in healthcare utilization and outcomes along socioeconomic lines. For example, an investigation at a major public hospital in Los Angeles revealed that Hispanic patients postponed care seeking and faced elevated mortality rates, both of which were significantly linked to lower community median incomes, reduced educational attainment, and greater concentrations of immigrants [32]. Despite interventions by local governments—including targeted health checkups, medication subsidies, and home-based nursing services for those at a disadvantage—significant gaps in care provision remained, and these disparities were amplified during the pandemic.

Regarding trends in medical expenditures, our analysis confirmed that patients with a USC exhibited the smallest increases in healthcare costs during the early pandemic period. Between 2019 and 2020, there were concerns about possible interruptions in chronic disease management due to restricted access to healthcare services. Nevertheless, increases in healthcare costs were relatively stable among those with a USC. Specifically, patients lacking a USC experienced a 55.4% rise in expenditures, those with only a usual place (but not a physician) had a 35.6% increase, while those with a usual physician at a specific location exhibited just a 3.6% increase. The utilization of telehealth likely played a role in maintaining continuity of diabetes management through this period by supporting consistent medication prescribing and glycemic monitoring [33]. Prior studies have also demonstrated that remote healthcare services can reduce the need for in-person visits while preserving continuity of care for chronic diseases, including diabetes [34, 35].

A U.S. study reported that while the incidence of diabetic foot ulcers and amputations remained stable during the pandemic, the rate of lower extremity amputations surged afterward. This pattern suggests that delayed interventions during the pandemic may have resulted in preventable complications [36]. In Korea, analysis of claims data from the NHI program suggests that patients cared for primarily at primary care clinics experience superior health outcomes—such as lower hospitalization and mortality risks—compared with those seen at secondary or tertiary hospitals [37].

Our findings also indicate that adult diabetes patients whose primary care physician was rated highly for comprehensiveness and coordination were associated with over 10% lower medical expenditures than those who did not have a physician as their USC. This finding supports established evidence regarding the advantages of integrated primary care models, which prioritize patient education, medication management, and prevention of complications as essential for improving outcomes and lowering expenditures [38, 39].

Importantly, the proportion of patients identifying hospital-based physicians as their USC decreased from 42.2% in 2019 to 30.3% in 2022, indicating persistent disparities in Korea’s healthcare system. This shift might reflect increased utilization of outpatient clinics during the pandemic, along with broader policy efforts to enhance community-based management of chronic diseases like diabetes and hypertension. Since Wagner’s Chronic Care Model was adopted in the United States, there has been heightened emphasis on coordinated and preventive care to improve cost-effectiveness, a focus that has been reinforced through incentives in programs such as Medicare [40–42]. Such approaches—including intermediate care, remote glucose monitoring, and telehealth—align with the role of USC in effective chronic condition management.

In Korea, private health insurance (PHI) is widespread and has been demonstrated to affect healthcare utilization and medical spending. Approximately 70–80% of Korean adults participate in some type of PHI, with higher enrollment rates particularly evident among those with higher incomes, advanced education, and those residing in urban areas [43]. In this study, we conducted a sensitivity analysis that included PHI enrollment status as an independent (exogenous) variable. The analysis showed that patients with a high-quality primary care physician as their USC had a 13.0% reduction in total medical expenditures (*β* =–0.139, *P* = 0.007), a result closely matching the 13.1% reduction observed in the main analysis.

We further employed a fixed-effects model analysis using the Fixone option in SAS 9.4 to evaluate the consistency of our results. However, this approach did not yield any statistically significant outcomes. This lack of significance may result from the limited within-individual variation in the type of USC during the four-year observation window, as well as the restriction of fixed-effects models in estimating coefficients for time-invariant covariates, including education level or insurance type. In contrast, the random-effects model and Hausman–Taylor estimator consistently demonstrated significant associations in the expected direction, with high-quality community clinic physicians as USC linked to lower expenditures (*β* = − 0.145, *P* = 0.005) and hospital-based USC tending toward higher expenditures (*β* = 0.096, *P* = 0.049; see Supplementary Table S1). Due to these constraints, the Hausman–Taylor estimation was considered more suitable, as it permits the inclusion of both time-varying and time-invariant variables while controlling for potential endogeneity. Taken together, the consistency in effect directions across all models supports the robustness of our primary findings.

This study has several limitations. First, while we compared healthcare costs by type of usual healthcare provider, the dataset lacked comprehensive details regarding care quality, glycemic control, or diabetes-related complication rates. In addition, our measure of USC quality relied solely on patient self-reports of comprehensiveness and coordination, which may be subject to reporting bias and limited validity. Second, indirect costs such as transportation, lost productivity, or caregiver responsibilities were not measured. Third, panel attrition surpassed 20% in the pandemic years. Although cross-sectional weights were applied in descriptive analyses, longitudinal weights were not incorporated in the regression models due to the use of a balanced panel restricted to individuals observed in all four survey years. Consequently, our findings are most representative of this consistently observed cohort, and some caution is warranted when generalizing results to the broader diabetic population in Korea. Fourth, the KHP’s chronic disease survey does not distinguish between type 1 and type 2 diabetes, restricting our analysis of these subgroups. However, given that type 1 diabetes represents less than 1% of adult diabetes cases in Korea based on national epidemiologic data [44], the effect of this limitation on our results is expected to be minimal.

This investigation is among the earliest to deliver nationally representative data on the association between having a usual source of care and healthcare expenditures before and throughout the COVID-19 pandemic in Korea.

## Conclusion

Korea has introduced a range of initiatives aimed at enhancing the quality of primary care for patients with diabetes [29]. With the challenges posed by an aging demographic and increasing healthcare expenditures, it is imperative to implement preventive measures and make systematic reforms to curb disease progression and associated complications [45]. Developing a primary care framework that is high-quality, comprehensive, and coordinated around a USC remains essential for long-term chronic disease management. In light of the current administration’s commitment to establishing a designated primary care physician system, our results—which show that having a high-quality primary care physician as a USC is associated with lower medical expenditures among adults with diabetes—offer pertinent evidence in favor of this policy. These findings underscore the ability of the proposed system to enhance healthcare efficiency and patient outcomes in the context of persistent public health issues.

## Supplementary Information

Below is the link to the electronic supplementary material.

Supplementary Material 1

## Data Availability

Data supporting the findings of our study are openly available on the Korea Health Panel at https://www.khp.re.kr:444/eng/main.do is available from the corresponding author upon request.
