# Supplementary material for: Effects of the type and quality of usual source of care on medical expenditures in adults with diabetes before and during the COVID‑19 pandemic: a panel data analysis using the Korea Health Panel (2019–2022)
Source: BMC Health Serv Res. 2025 Oct 15;25:1369. doi: 10.1186/s12913-025-13518-7 (PMC12522456; doi:10.1186/s12913-025-13518-7)
Supplement: Supplementary file 1 — Supplementary Material 1 [file 12913_2025_13518_MOESM1_ESM.docx]

Supplementary Table S1. **Robustness checks using fixed-effects and random-effects panel models** (Dependent variable: log-transformed total medical expenditures)

|  |  | | Fixed-effects | | | Random-effects | | |
| --- | --- | --- | --- | --- | --- | --- | --- | --- |
|  |  | | Estimate | SE | *P*-value | Estimate | SE | *P*-value |
| Age group (ref: 19–49 years) | 50–64 years | | -0.173 | 0.303 | 0.568 | 0.104 | 0.128 | 0.417 |
|  | 65 years or older | | -0.184 | 0322 | 0.568 | 0.192 | 0.128 | 0.134 |
| Sex (ref: male) | Female | | Not available | | | -0.046 | 0.056 | 0.408 |
| Education level (ref: elementary or less) | Middle to high school | | Not available | | | 0.038 | 0.059 | 0.515 |
|  | College or higher | | Not available | | | 0.008 | 0.094 | 0.930 |
| Marital status (ref: never married) | Divorced, widowed, or separated | | 1.584 | 0.976 | 0.105 | -0.327 | 0.171 | 0.056 |
|  | Married | | 1.478 | 0.952 | 0.121 | -0.346 | 0.167 | 0.038 |
| Equalized personal income quintile ^~~a~~^ (ref: first) | Second | | 0.141 | 0.063 | 0.025 | 0.084 | 0.050 | 0.092 |
|  | Third | | 0.100 | 0.084 | 0.236 | 0.072 | 0.062 | 0.247 |
|  | Fourth | | 0.126 | 0.097 | 0.195 | 0.051 | 0.069 | 0.462 |
|  | Fifth | | 0.228 | 0.124 | 0.065 | 0.046 | 0.081 | 0.574 |
| Health insurance type (ref: employee) | Regional | | 0.115 | 0.095 | 0.228 | 0.095 | 0.049 | 0.053 |
|  | Medical Aid | | 0.066 | 0.222 | 0.767 | 0.101 | 0.100 | 0.317 |
| CCI score (ref: 1) | 2 or more | | -0.030 | 0.178 | 0.867 | 0.465 | 0.092 | < 0.001 |
| Number of healthcare utilizations (log-transformed) |  | | 1.230 | 0.041 | < 0.001 | 1.178 | 0.028 | < 0.001 |
| Subjective health status (ref: good) | Moderate | | 0.077 | 0.056 | 0.169 | 0.113 | 0.048 | 0.019 |
|  | Poor | | 0.173 | 0.064 | 0.007 | 0.288 | 0.053 | < 0.001 |
| Type of physician as a USC (ref: no usual physician) | PCP | High in quality | -0.012 | 0.060 | 0.839 | **-0.145** | **0.051** | **0.005** |
|  |  | Not high in quality | 0.005 | 0.055 | 0.923 | -0.087 | 0.047 | 0.066 |
|  | Hospital physician | | -0.037 | 0.057 | 0.514 | **0.096** | **0.048** | **0.047** |
| Survey year (ref: 2019) | 2020 | | 0.079 | 0.044 | 0.073 | 0.073 | 0.043 | 0.084 |
|  | 2021 | | 0.028 | 0.045 | 0.530 | 0.023 | 0.043 | 0.593 |
|  | 2022 | | 0.178 | 0.047 | < 0.001 | 0.178 | 0.044 | < 0.001 |
| R-square | | | 0.700 | | | 0.351 | | |
| Panel regression analyses were conducted using the fixone and ranone options of proc panel in SAS 9.4, based on a balanced panel derived from the KHP (2019–2022). The key independent variable was having a high-quality PCP as the USC. The definition of “high in quality” was a USC physician whom the patient rated positively (i.e., ‘good’ or ‘very good’) for both comprehensiveness and coordination functions. Individuals with no healthcare use in the previous year or who indicated a public health center or herbal clinic as their USC were excluded from the study. KHP: Korea Health Panel; USC: usual source of care; PCP: primary care physician; CCI: Charlson Comorbidity Index. | | | | | | | | |
